# Burnout and Eating-Disorder Screening Risk Among Health Sciences Students in Peru: A Cross-Sectional Study

**DOI:** 10.3390/healthcare14162616

**Published:** 2026-08-19

**Authors:** Samantha Sotelo-Llancari, Carlos J. Zumaran-Nuñez, Liseth Pinedo-Castillo

**Affiliations:** 1 Universidad Autónoma del Perú, Lima 150142, Peru; 2 Universidad Señor de Sipán, Chiclayo 14001, Peru; calin-peru2@hotmail.com (C.J.Z.-N.); lisethpinedo99@gmail.com (L.P.-C.)

**Keywords:** burnout, eating-disorder screening, health sciences students, university students, Peru

## Abstract

**Background/Objectives:** Burnout and eating-disorder screening risk are relevant concerns among health sciences students, but evidence from northern Peru remains limited. The primary objective was to examine whether an archived study-specific burnout classification (independent/explanatory variable) was associated with probable eating-disorder symptomatology (dependent/outcome variable) among students enrolled in Human Medicine, Nursing, and Dentistry. **Methods:** A cross-sectional study was conducted at a private university in the Lambayeque Region, Peru, during the 2023-II academic semester. The analytical sample comprised 200 students. Eating-disorder screening was assessed with the Eating Attitudes Test-26 (EAT-26), and burnout was assessed with a 22-item Maslach Burnout Inventory (MBI) version. The revision uses retained aggregate outputs because individual-level item data are no longer available. **Results:** Seventy-four students (37.0%) were in the archived probable eating-disorder symptomatology category. The corresponding proportions were 0/53 (0.0%) in the study-specific low MBI category, 21/80 (26.3%) in the medium category, and 53/67 (79.1%) in the high category (chi-square = 86.05, *p* < 0.001; Cramer’s V = 0.656). Exploratory differences were also observed by sex, living arrangement, academic program, employment status, study time, and sleep duration. **Conclusions:** The archived categorical results show a strong bivariate association between the two study-specific screening classifications. Because the design was cross-sectional and non-probabilistic and the item-level data needed to verify instrument scoring and reliability are unavailable, the findings should not be interpreted as causal or diagnostic.

## 1. Introduction

Burnout in health sciences education is commonly conceptualized through exhaustion, psychological distancing or depersonalization, and reduced efficacy or accomplishment, although the operational definition varies across instruments and study populations [1]. Studies among medical, nursing, and other health sciences students have associated burnout indicators with academic workload, stage of training, sleep, engagement, employment, and program characteristics [2,3,4,5,6]. These findings support examining burnout as a student well-being concern within demanding training environments while avoiding direct transfer of occupational interpretations to university students.

Eating-disorder screening risk is also relevant in medical and health sciences students. A systematic review and meta-analysis found substantial heterogeneity in eating-disorder symptomatology across medical-student samples [7]. Recent studies have linked eating-disorder risk or symptoms with sex, perceived stress, sleep difficulties, psychological distress, body-related pressures, financial hardship, and adverse experiences during training [8,9,10,11]. Screening instruments identify students who may require further assessment; they do not establish a clinical diagnosis.

The potential co-occurrence of burnout-related distress and disordered-eating symptoms is theoretically plausible but should be interpreted cautiously. Transdiagnostic models of eating disorders emphasize cognitive and behavioral processes that may be intensified under distress [12]. In student populations, perceived stress, restricted sleep, academic pressure, and reduced engagement are correlates reported across the burnout and eating-disorder literatures [3,4,5,8,11]. These overlapping correlates provide a rationale for examining the two constructs together, but the present study did not directly measure mediating mechanisms such as emotion regulation, perfectionism, interoception, or attachment; therefore, no mechanistic pathway is tested here.

Peruvian evidence remains comparatively limited. In a study of first-year medical students in Lima, 10.1% met the EAT-26 threshold used for probable eating-disorder symptomatology [13]. Evidence from northern Peru and from samples that include multiple health sciences programs is less represented. Examining students from Human Medicine, Nursing, and Dentistry may therefore add descriptive evidence from a setting that is underrepresented in the international literature.

Accordingly, the primary objective of this study was to examine the association between the archived study-specific burnout classification and probable eating-disorder symptomatology among health sciences students in Peru. For the primary analysis, probable eating-disorder symptomatology was treated as the dependent/outcome variable and the study-specific MBI category as the independent/explanatory variable. Secondary analyses explored the distribution of the outcome according to sociodemographic characteristics (sex, age, marital status, place of origin, monthly income, and living arrangement), academic characteristics (program and daily study time), employment characteristics (employment status and work time), and sleep duration. We hypothesized that probable eating-disorder symptomatology would be more frequent in higher study-specific MBI categories.

## 2. Materials and Methods

### 2.1. Study Design and Setting

This observational, cross-sectional study was conducted among health sciences students at a private university in the Lambayeque Region of northern Peru. Data collection began only after institutional approval was issued on 3 October 2023 and was conducted during the 2023-II academic semester through an online questionnaire. The exact final calendar date of data collection was not preserved in the retained study documentation.

### 2.2. Study Population, Sample, Sampling, and Eligibility

The target population comprised 1013 students (*N* = 1013) enrolled in the relevant academic stages of Human Medicine, Nursing, and Dentistry. The original minimum sample size was calculated with a finite-population formula using a 95% confidence level, a 5% margin of error, and an expected prevalence of 10.10%, based on the Peruvian study by Ponce Torres et al. [13]; this produced a minimum of 123 participants. A total of 206 students completed the survey, and 200 were retained in the analytical sample (*n* = 200) after six records were excluded. The analytical sample represented 19.7% of the target population. Because the number of students who actually received an invitation was not retained by program, this proportion should not be interpreted as a program-specific response rate.

Sampling was non-probabilistic and participation was voluntary. Students were eligible if they were regularly enrolled, provided informed consent, and were in the 11th or 12th cycle of Human Medicine or the 7th or 8th cycle of Nursing or Dentistry. Students were excluded if they were completing a medical internship, were outside the specified academic cycles, were not enrolled at the participating university in one of the three selected programs, or submitted incomplete, implausible, or internally inconsistent responses. The archived materials confirm that invitations led to an online survey but do not preserve the specific recruitment channel used to distribute the invitation.

The expected prevalence used in the original sample-size calculation (10.10%) was lower than the archived observed proportion in the probable eating-disorder symptomatology category (37.0%). If 37.0% were used in the same finite-population formula with a 5% margin of error, the required sample would be approximately 265 students. Accordingly, prevalence estimates in this sample should be interpreted as descriptive rather than as estimates with the originally intended precision.

### 2.3. Study Variables

The primary dependent/outcome variable was probable eating-disorder symptomatology according to the archived EAT-26 classification. For the primary association analysis, the outcome was represented as probable symptomatology (yes/no). The primary independent/explanatory variable was the archived study-specific MBI category (low, medium, or high). Secondary variables were grouped as follows: sociodemographic variables included sex, age, marital status, place of origin, monthly income, and living arrangement (living with parents or not living with parents); academic variables included academic program and daily study time; employment variables included employment status (not employed while studying or employed while studying) and work time; and the sleep-related variable was nightly sleep duration.

### 2.4. Psychometric Instruments and Archived Scoring

Eating-disorder screening was performed with the 26-item Eating Attitudes Test (EAT-26), developed by Garner, Olmsted, Bohr, and Garfinkel [14]. The instrument includes items related to dieting, bulimia/food preoccupation, and oral control. In the conventional scoring approach, item responses are recoded to yield a total score from 0 to 78, and a score of 20 or higher is commonly used as a screening threshold warranting further assessment [14]. A Spanish-language validation by Rivas et al. reported high internal consistency for the total EAT-26, with Cronbach’s alpha coefficients of 0.904 in a community sample (*n* = 778) and 0.938 in a case–control sample (*n* = 156) [15]. The original study additionally divided scores below 20 into two archived categories (1–9 and 10–19). These lower-score categories are therefore treated in this revision as study-specific descriptive strata rather than as standard diagnostic categories.

Burnout was assessed in the original study using a 22-item Maslach Burnout Inventory (MBI) version. The MBI framework developed by Maslach, Jackson, and Leiter assesses emotional exhaustion, depersonalization, and personal accomplishment as distinct dimensions [16]. Because the MBI is multidimensional, internal consistency is appropriately reported by subscale rather than as a single total-scale coefficient. Published psychometric evidence for the Spanish 22-item adaptation reports Cronbach’s alpha coefficients of 0.90 for emotional exhaustion, 0.79 for depersonalization, and 0.71 for personal accomplishment [17]. Standard interpretation emphasizes these subscales rather than a single summed clinical score. However, the archived original analysis created a total score ranging from 0 to 132 and classified it as low (0–43), medium (44–88), or high (89–132). In this revision, those categories are retained only as study-specific exposure strata and are not presented as validated clinical cutoffs.

The individual item-level dataset is no longer available to the authors. The original undergraduate thesis describing the study is publicly archived [18] and confirms the use of the EAT-26 and the 22-item MBI; however, it does not contain the participant-level item responses required to recompute sample-specific internal-consistency coefficients. Consequently, the exact EAT-26 item recoding applied in the original analysis cannot be independently verified, MBI subscale scores cannot be reconstructed, and Cronbach’s alpha, McDonald’s omega, or other current-sample reliability coefficients cannot be calculated retrospectively. The validation coefficients reported above therefore describe published Spanish-language psychometric evidence and should not be interpreted as reliability estimates from the present sample. For the same reason, the revised Section 3 omits the previously reported continuous EAT-26 and composite MBI summary scores and limits interpretation to the retained categorical contingency tables.

### 2.5. Data Collection and Missing-Data Information

Data were obtained through an online questionnaire that contained the study variables and the two psychometric instruments. Six of the 206 submitted records were excluded because they failed eligibility or completeness/consistency criteria, leaving 200 records in the archived analytical outputs. The retained marginal tables sum to *n* = 200 for the reported participant characteristics, suggesting that the final tabulations were based on complete category-level records for those variables; however, item-level missingness cannot be independently verified without the original dataset. The detailed cross-tabulation of work-time categories by eating-disorder outcome was not retained and is therefore not reproduced in the revised comparative table.

### 2.6. Statistical Analysis

Categorical variables are reported as frequencies and percentages. Wilson 95% confidence intervals are provided for the main archived screening-category proportions. The association between the study-specific MBI category and probable eating-disorder symptomatology was defined as the primary hypothesis and was evaluated from the retained 3 × 2 contingency table using Pearson’s chi-square test. Cramer’s V was recomputed from the archived cell counts as an effect-size measure. Secondary comparisons between probable eating-disorder symptomatology and participant characteristics are treated as exploratory. Their *p* values are those retained from the original chi-square or Fisher’s exact analyses, while Cramer’s V was recomputed from the available contingency counts. Because one primary hypothesis was distinguished a priori from the exploratory comparisons, no family-wise multiplicity adjustment is used for the secondary analyses, and these *p* values are interpreted cautiously rather than as confirmatory evidence of independent effects.

No multivariable model can be reconstructed from the available aggregate outputs; therefore, the study cannot distinguish independent associations from confounding among correlated student characteristics. Continuous-score comparisons that depended on unverified EAT-26 or composite MBI scoring were removed from the revised manuscript. Reporting was revised with reference to the STROBE recommendations for observational studies, to the extent permitted by the retained study documentation [19].

### 2.7. Ethical Considerations

The institutional review body for this study was the Research Committee of the Professional School of Human Medicine, Faculty of Health Sciences, Universidad Señor de Sipán. The research project received institutional approval through Faculty Resolution No. 0583-2023/FCS-USS, dated 3 October 2023. Under the institutional research procedures in force at the time, student research projects were reviewed by the School Research Committee before issuance of the corresponding Faculty resolution. Data collection began only after this approval. Participation was voluntary, and electronic informed consent was obtained before access to the questionnaire. Responses were handled using numerical identifiers in the analytical materials. The study was conducted in accordance with the Declaration of Helsinki [20]. Subsequent Faculty resolutions issued in March 2024 documented administrative updates to the same research project, including updates to authorship and the project title, and did not represent initiation of a new study or a new data-collection phase.

## 3. Results

### 3.1. Participant Flow and Sample Characteristics

Of 206 students who submitted the survey, six records were excluded because they did not meet the eligibility or completeness/consistency criteria. The final analytical sample comprised 200 students. Most participants were female (75.5%), aged 18–30 years (79.0%), single (77.0%), and from Chiclayo (89.0%). Slightly more than half lived with their parents (53.0%). Nursing represented 42.0% of the sample, Human Medicine 34.5%, and Dentistry 23.5%. Forty-five percent were employed while studying, 38.0% reported studying at least six hours per day, and 84.5% reported sleeping seven hours or less per night (Table 1).

### 3.2. Archived Instrument Classifications

The archived EAT-26 classification placed 68 students (34.0%) in the no-risk stratum, 58 (29.0%) in the intermediate study-specific risk stratum, and 74 (37.0%; 95% CI 30.6–43.9%) in the probable eating-disorder symptomatology category. For the archived study-specific MBI classification, 53 students (26.5%) were classified as low, 80 (40.0%) as medium, and 67 (33.5%) as high (Table 2). As specified in the Methods section, continuous instrument scores and MBI subscale results are not reported because the item-level scoring cannot be reconstructed from the retained materials.

### 3.3. Primary Association Between the Archived Burnout and Eating-Disorder Classifications

The primary contingency table showed a marked difference in probable eating-disorder symptomatology across the study-specific MBI categories. No student in the low category (0/53) was in the probable symptomatology category, compared with 21/80 (26.3%) in the medium category and 53/67 (79.1%) in the high category. The association was statistically significant (Pearson chi-square = 86.05, df = 2, *p* < 0.001) and the effect size calculated from the retained counts was large (Cramer’s V = 0.656). These figures describe between-group differences at one time point and do not represent an increase over time (Table 3).

### 3.4. Exploratory Comparisons by Participant Characteristics

Exploratory comparisons showed that probable eating-disorder symptomatology was more frequent among female students than male students (44.4% vs. 14.3%), among students who did not live with their parents than those who did (61.7% vs. 15.1%), among students employed while studying than those not employed (53.3% vs. 23.6%), and among students studying at least six hours per day than those studying less than six hours (59.2% vs. 23.4%). The proportion was also higher among students reporting seven hours of sleep or less (41.4%) than among those reporting more than seven hours (12.9%). Differences were observed across monthly income and academic-program categories, whereas age, marital status, and place of origin did not show statistically significant differences in the retained original analyses. These comparisons are exploratory and unadjusted for confounding (Table 4).

## 4. Discussion

The principal finding was a strong bivariate association between the archived study-specific MBI category and probable eating-disorder symptomatology. Probable symptomatology was observed in 0 of 53 students in the low MBI category, 21 of 80 in the medium category, and 53 of 67 in the high category. The large Cramer’s V indicates substantial separation between the archived categories in this sample. Nevertheless, this association should be interpreted as a cross-sectional pattern rather than evidence that burnout causes eating-disorder symptoms or vice versa. In addition, the original MBI composite categorization and the EAT-26 scoring cannot be independently verified from item-level data, which limits clinical interpretation of the gradient.

The direction of the observed association is broadly compatible with the literature showing that psychological distress, stress, and eating-related symptoms can cluster in health sciences students. Bounar et al. examined eating-disorder-related behaviors together with stress, burnout, and other mental-health problems among medical students [10], while Okasha et al. reported an association between perceived stress and eating-disorder risk in university students [11]. Meta-analytic evidence also confirms that eating-disorder symptomatology is a relevant concern in medical-student populations [7]. These studies support the plausibility of co-occurrence, but they do not establish a single causal mechanism linking burnout and disordered eating.

The exploratory findings also warrant cautious comparison with previous research. Female students had a higher proportion of probable eating-disorder symptomatology than male students, consistent with findings reported in medical-student samples in Romania and France [8,9]. Differences across academic programs were also observed, but the present study cannot determine whether they reflect program-specific demands, sex composition, employment patterns, or other confounding factors. Accordingly, program comparisons should be treated as descriptive rather than as evidence that one program independently confers greater risk.

Living arrangement, employment while studying, study time, and sleep duration were also associated with the outcome in unadjusted analyses. Student-burnout studies have linked sleep, workload, engagement, and academic context to burnout indicators [3,4,5,6], and eating-disorder studies have identified sleep and stress-related correlates [8,11]. In the present data, however, living away from parents cannot be equated with reduced social support, and employment cannot be assumed to cause time scarcity or psychological distress because those constructs were not directly measured. These variables are best interpreted as contextual characteristics that identify subgroups with different observed distributions of the screening outcome.

Several limitations materially constrain interpretation. First, the study was cross-sectional and used non-probabilistic voluntary sampling, which limits temporal inference and generalizability and introduces possible selection bias. Second, the original sample-size calculation used an expected prevalence of 10.1%, whereas the archived observed probable-symptomatology proportion was 37.0%; under the same precision assumptions, a prevalence of 37.0% would require a larger sample. Third, the individual-level and item-level dataset is no longer available. Consequently, EAT-26 scoring cannot be rechecked, MBI subscales cannot be reconstructed, current-sample reliability cannot be estimated, and multivariable models or sensitivity analyses cannot be performed. Fourth, the archived MBI total-score categories are not standard subscale-based MBI interpretations and are therefore treated only as study-specific strata. Fifth, the secondary comparisons are exploratory and unadjusted for confounding and multiple testing. Sixth, the exact final date of data collection, the detailed invitation procedure, and the number of students actually invited by program were not retained. These limitations require cautious interpretation of the observed associations and prevalence-like proportions.

Within these constraints, the findings support the value of future prospective studies that retain item-level data, use a student-appropriate and fully specified burnout instrument, report subscale scores and reliability, apply validated EAT-26 scoring, document recruitment and response rates by program, and use multivariable models to evaluate independent associations. Universities may also consider accessible mental-health and nutrition services for students who report distress, while screening results should always be linked to appropriate clinical assessment rather than treated as diagnoses.

## 5. Conclusions

Among 200 health sciences students in northern Peru, the archived study-specific burnout classification was strongly associated with probable eating-disorder symptomatology in a bivariate contingency analysis. Probable symptomatology was present in 0.0% of the low, 26.3% of the medium, and 79.1% of the high study-specific MBI categories, with a large effect size. Exploratory differences were also observed across sex, living arrangement, academic program, employment status, study time, and sleep duration.

The findings are descriptive and should be interpreted with substantial methodological caution. The cross-sectional non-probabilistic design, lack of individual-level data, inability to verify EAT-26 scoring, nonstandard archived MBI composite categories, absence of current-sample reliability and subscale analyses, and inability to conduct multivariable adjustment prevent causal or diagnostic conclusions. Future studies with prospectively retained data and standardized psychometric scoring are needed to determine whether the association is robust and independent of other student characteristics.

## Figures and Tables

**Table 1:** Sociodemographic, academic, employment, and sleep-related characteristics of the analytical sample (*n* = 200).

%	*n*	Characteristic
		Sex
75.50	151	Female
24.50	49	Male
		Age (years)
79.00	158	18–30
20.00	40	31–59
1.00	2	≥60
		Marital status
77.00	154	Single
21.00	42	Married/Cohabiting
2.00	4	Widowed/Divorced
		Place of origin
89.00	178	Chiclayo
11.00	22	Other provinces
		Monthly income (PEN)
17.50	35	>5000
51.00	102	1500–4999
19.50	39	1025–1500
12.00	24	<1025
		Living arrangement
53.00	106	Lives with parents
47.00	94	Does not live with parents
		Academic program
34.50	69	Human Medicine
42.00	84	Nursing
23.50	47	Dentistry
		Employment status
55.00	110	Not employed while studying
45.00	90	Employed while studying
		Daily study time
62.00	124	<6 h
38.00	76	≥6 h
		Work time
51.50	103	Does not work
39.00	78	Part-time
9.50	19	Full-time
		Nightly sleep duration
84.50	169	≤7 h
15.50	31	>7 h

**Table 2:** Archived study-specific EAT-26 and MBI classifications (*n* = 200).

95% CI for %	%	*n*	Archived Classification	Instrument
27.8–40.8	34.0	68	No-risk stratum	EAT-26
23.2–35.6	29.0	58	Intermediate study-specific risk stratum	
30.6–43.9	37.0	74	Probable eating-disorder symptomatology	
20.9–33.0	26.5	53	Study-specific low category	MBI
33.5–46.9	40.0	80	Study-specific medium category	
27.3–40.3	33.5	67	Study-specific high category	

Note: The EAT-26 categories below the conventional threshold of 20 and the MBI low/medium/high composite categories reflect the archived original analysis and are treated as study-specific descriptive strata. They are not presented as validated diagnostic or clinical cutoffs. CI: confidence interval; EAT-26: Eating Attitudes Test-26; MBI: Maslach Burnout Inventory.

**Table 3:** Primary association between study-specific MBI category and probable eating-disorder symptomatology (*n* = 200).

95% CI for %	Probable Symptomatology, %	Other EAT-26 Strata, *n*	Probable Symptomatology, *n*	Study-Specific MBI Category
0.0–6.8	0.00	53	0	Low
17.9–36.8	26.25	59	21	Medium
67.9–87.1	79.10	14	53	High

Note: Pearson chi-square = 86.05, df = 2, *p* < 0.001; Cramer’s V = 0.656. Percentages are calculated within each study-specific MBI category. EAT-26: Eating Attitudes Test-26; MBI: Maslach Burnout Inventory.

**Table 4:** Exploratory distribution of probable eating-disorder symptomatology by participant characteristics.

Cramer’s V	*p* Value *	Probable, %	Probable, *n*/*N*	Characteristic
0.268	<0.001			Sex
		44.37	67/151	Female
		14.29	7/49	Male
0.085	0.692			Age (years)
		37.97	60/158	18–30
		35.00	14/40	31–59
		0.00	0/2	≥60
0.037	1.000			Marital status
		37.01	57/154	Single
		38.10	16/42	Married/Cohabiting
		25.00	1/4	Widowed/Divorced
0.281	0.001			Monthly income (PEN)
		14.29	5/35	>5000
		34.31	35/102	1500–4999
		53.85	21/39	1025–1500
		54.17	13/24	<1025
0.062	0.384			Place of origin
		35.96	64/178	Chiclayo
		45.45	10/22	Other provinces
0.482	<0.001			Living arrangement
		15.09	16/106	Lives with parents
		61.70	58/94	Does not live with parents
0.273	0.001			Academic program
		37.68	26/69	Human Medicine
		48.81	41/84	Nursing
		14.89	7/47	Dentistry
0.306	<0.001			Employment status
		23.64	26/110	Not employed while studying
		53.33	48/90	Employed while studying
0.360	<0.001			Daily study time
		23.39	29/124	<6 h
		59.21	45/76	≥6 h
0.214	0.003			Nightly sleep duration
		41.42	70/169	≤7 h
		12.90	4/31	>7 h

Note: * *p* values are the retained *p* values from the original chi-square or Fisher’s exact analyses. Cramer’s V values were recomputed from the archived contingency counts using Pearson chi-square counts and are provided as descriptive effect sizes. The work-time comparison is omitted because its detailed outcome cross-tabulation was not retained, although the marginal work-time distribution is shown in Table 1.

## Data Availability

The individual-level dataset and item-level psychometric responses are no longer available to the authors. The revised analyses are therefore limited to retained aggregate contingency tables and statistical outputs. The original undergraduate thesis describing the study is publicly available in the Universidad Señor de Sipán repository [19]. The thesis documents the study but does not restore access to the underlying item-level response data needed for retrospective reliability estimation. Because individual-level data are unavailable, additional psychometric, reliability, multivariable, or sensitivity analyses cannot be reproduced.
